# A Mechanistic Review of Mitophagy and Its Role in Protection against Alcoholic Liver Disease

**DOI:** 10.3390/biom5042619

**Published:** 2015-10-16

**Authors:** Jessica A. Williams, Wen-Xing Ding

**Affiliations:** Department of Pharmacology, Toxicology and Therapeutics, University of Kansas Medical Center, 3901 Rainbow Blvd., Kansas City, KS 66160, USA; E-Mail: jesswilliams888@gmail.com

**Keywords:** alcohol, mitochondria, mitophagy, autophagy, Parkin, liver injury

## Abstract

Alcoholic liver disease (ALD) is a major health problem worldwide, and alcohol is well-known to cause mitochondrial damage, which exacerbates alcohol-induced liver injury and steatosis. No successful treatments are currently available for treating ALD. Therefore, a better understanding of mechanisms involved in regulation of mitochondrial homeostasis in the liver and how these mechanisms may protect against alcohol-induced liver disease is needed for future development of better therapeutic options for ALD. Mitophagy is a key mechanism for maintaining mitochondrial homeostasis by removing damaged mitochondria, and mitophagy protects against alcohol-induced liver injury. Parkin, an E3 ubiquitin ligase, is well-known to induce mitophagy in *in vitro* models although Parkin-independent mechanisms for mitophagy induction also exist. In this review, we discuss the roles of Parkin and mitophagy in protection against alcohol-induced liver injury and steatosis. We also discuss Parkin-independent mechanisms for mitophagy induction, which have not yet been evaluated in the liver but may also potentially have a protective role against ALD. In addition to mitophagy, mitochondrial spheroid formation may also provide a novel mechanism of protection against ALD, but the role of mitochondrial spheroids in protection against ALD progression needs to be further explored. Targeting removal of damaged mitochondria by mitophagy or inducing formation of mitochondrial spheroids may be promising therapeutic options for treatment of ALD.

## 1. Introduction

Alcoholic liver disease (ALD) is a global health problem that is caused by heavy alcohol consumption in addition to other environmental, lifestyle, and genetic factors. ALD causes approximately two million deaths per year [1], and alcohol abuse leads to a variety of liver pathologies including fatty liver, alcoholic hepatitis, fibrosis, cirrhosis, and hepatocellular carcinoma (HCC) [2,3].

The best therapeutic options for ALD patients are abstinence from alcohol or liver transplantation. Abstaining from alcohol can reverse ALD in early disease states and can also decrease mortality rates [4]. Other therapies are used to treat ALD patients such as corticosteroids and tumor necrosis factor alpha (TNFα) inhibitors, but they are not successful for all patients [5,6,7]. Therefore, liver transplantation is currently the best option for decreasing mortality in ALD. However, the option for liver transplantation is only given to those with severe disease and/or to patients who have been sober for at least 6 months [8]. Therefore, a better understanding of the mechanisms underlying ALD pathogenesis and progression is greatly needed.

Macroautophagy (hereafter referred to as autophagy) is a protective and evolutionarily conserved process that degrades various macromolecules in the lysosome [9], and it can be both a selective and non-selective process. Non-selective autophagy, also known as bulk autophagy, occurs during starvation to provide amino acids that can be used for energy production [10]. In addition, degradation of lipids by autophagy produces free fatty acids that can be used to make energy by β-oxidation in the mitochondria [11]. Selective autophagy removes damaged organelles and protein aggregates using specific receptors, and it can occur in both nutrient-rich and poor conditions [12]. The role of autophagy in ALD has been recently reviewed [3,13,14,15,16,17]. We and others have shown that autophagy protects against alcohol-induced liver injury [13,18,19], and protection is due to selective removal of damaged mitochondria and lipid droplets by mitophagy and lipophagy, respectively [13,18]. Mitophagy is a form of selective autophagy specific for degradation of damaged mitochondria in the lysosome [20,21,22], and lipophagy is a form of selective autophagy specific for the degradation of lipid droplets [11,23,24,25]. Regulation of the lipophagy pathway is not yet understood but may require the GTPase Rab7 [26]. The role of mitophagy in protection against ALD and mechanisms for its regulation are the main foci of this review.

Mitochondria are the major sites of ATP production and are also involved in heme synthesis, fatty acid oxidation, maintenance of calcium levels, and initiation of cell death [27], and mitochondrial dysfunction leads to progression of ALD [28,29,30]. Mitochondria maintain their homeostasis by various mechanisms. Mitochondria have their own proteolytic system, which degrades misfolded proteins [31,32]. Mitochondria also utilize the proteasome to degrade damaged outer mitochondrial membrane proteins [33]. In addition, damaged mitochondria can be segregated from healthy mitochondria by mitochondrial fission, and healthy mitochondria can combine their components by mitochondrial fusion [34,35]. Moreover, mitochondria can degrade oxidized proteins via mitochondria-derived vesicles, which bud off damaged mitochondria and are degraded in the lysosome along with their contents [36,37]. Furthermore, mitophagy degrades damaged mitochondria in the lysosome [20,21,22,38], which is an important mechanism for protection against alcohol-induced liver injury and steatosis [13,18,39]. Formation of mitochondrial spheroids may serve as an alternate pathway for removal of damaged mitochondria [38,40,41,42] and may also protect against alcohol-induced liver injury. Mitophagy and formation of mitochondrial spheroids as protective mechanisms against alcohol-induced liver injury and steatosis are discussed in this review.

## 2. Alcohol Metabolism

Alcohol metabolism has been extensively reviewed previously [43,44,45]. Briefly, alcohol metabolism occurs mainly in the liver, and alcohol is metabolized by both oxidative and non-oxidative pathways. Oxidative pathways are the predominant mechanism for alcohol metabolism. The most common pathway for oxidative metabolism in the liver is catalyzed by alcohol dehydrogenase (ADH), which metabolizes alcohol into acetaldehyde. Alcohol can also be oxidized into acetaldehyde by Cytochrome P450 2E1 (CYP2E1) and catalase [43,44]. Acetaldehyde is a highly reactive metabolite that forms adducts with other macromolecules. Proteins adducted by acetaldehyde have altered function, which sometimes results in loss of activity and subsequent liver injury [46]. Acetaldehyde is further metabolized into acetate and acetyl-CoA for use in metabolic pathways by aldehyde dehydrogenase (ALDH), which has two isoforms: cytosolic ALDH1 or mitochondrial ALDH2. Alcohol metabolism by ADH and ALDH require reduction of NAD^+^ to NADH. Therefore, oxidative alcohol metabolism results in a decreased NAD^+^/NADH ratio, leading to down-regulation of metabolic pathways that require NAD^+^ as a co-factor [43,44]. In addition, metabolism of alcohol by CYP2E1 results in production of reactive oxygen species (ROS), which leads to liver injury [47,48]. For non-oxidative alcohol metabolism, alcohol is esterified to fatty acid ethyl esters(FAEEs) by FAEE synthase or alcohol interacts with Phospholipase D to form phosphatidyl ethanol [44,45]. FAEEs may exacerbate alcohol-induced liver injury because they increase alcohol-induced cell death in HepG2 cells [49], but the effect of phosphatidyl ethanol formation in the liver is currently unknown.

## 3. ALD Pathogenesis

The first stage of ALD is hepatic steatosis, which is characterized as an abundance of fat in hepatocytes and develops in most heavy alcohol consumers. Steatosis is reversible with a few weeks of abstaining from alcohol, but it can further develop into steatohepatitis, fibrosis, cirrhosis, and even HCC with prolonged alcohol abuse in combination with other factors such as race, sex, comorbidities, genetics, and lifestyle factors [2]. Alcoholic steatohepatitis is characterized by hepatic inflammation and injury in addition to steatosis and also includes fibrotic and cirrhotic disease states. Most patients who stop using alcohol once cirrhosis has developed have a survival time of approximately 5 years after diagnosis [8,50,51]. Approximately 20% of all cases of liver cirrhosis in the United States are related to alcohol abuse [52], and an estimated 5 million people in the US are thought to have alcohol-induced steatohepatitis [53]. Cirrhosis is the end stage of ALD, and it is characterized by massive fibrosis and the presence of regenerative nodules in the liver in addition to loss of liver function. Fibrosis is an exacerbated wound-healing response induced by liver injury that is characterized by an accumulation of the extracellular matrix protein collagen, which is produced predominantly by hepatic stellate cells. Continuous activation of this wound healing response leads to cirrhosis of the liver, which can eventually progress to HCC [8,50,51,54].

In addition to heavy alcohol consumption, several other factors contribute to progression of ALD from steatosis to more severe liver pathologies including race, sex, lifestyle, drinking pattern, genetics, and comorbidities with other diseases. African-Americans and Hispanics are more prone to alcohol-induced cirrhosis than Caucasians [55,56], and women are more likely to progress beyond alcohol-induced steatosis compared to men after prolonged alcohol consumption [57,58,59]. Comorbidity with obesity [60,61,62,63] or Hepatitis C Virus [64,65,66] also increases ALD severity and promotes disease progression. Genetic polymorphisms and epigenetics also contribute to ALD progression and severity. Polymorphisms in ADH2, ADH3, ALDH2, and CYP2E1 [67,68] enhance ALD progression, and alcohol alters epigenetics and modifies histones in the GI tract and liver, which increases ALD severity [69]. Finally, lifestyle choices and drinking patterns affect ALD progression. For example, smoking exacerbates, while drinking coffee protects against, progression to liver cirrhosis [70,71,72,73,74]. The amount and duration of alcohol use in addition to the type of alcohol consumed and the pattern of alcohol consumption all also have roles in progression of ALD. Women who drink more than 40 grams of alcohol per day and men who drink more than 80 grams per day are more likely to develop ALD, but some may develop ALD by consuming less amounts of alcohol. In addition, wine drinkers may be less likely than beer or liquor drinkers to develop alcoholic cirrhosis, but this is controversial. Heavy daily drinking also increases the risk for developing ALD in comparison to weekly binge drinking [54].

## 4. Mitophagy Protects against Alcohol-Induced Liver Injury and Steatosis by Selectively Removing Damaged Mitochondria

We and others have shown that autophagy protects against alcohol-induced liver injury, as previously discussed. Acute alcohol treatment induces autophagy as a protective mechanism in mice and primary cultured mouse hepatocytes, and autophagy protects by selectively removing damaged mitochondria by mitophagy. In addition, inhibition of autophagy exacerbates ethanol-induced liver injury and cell death [18]. Moreover, autophagy activation protects against chronic alcohol-induced liver injury in mice fed a 4-week Lieber DeCarli alcohol diet, and it was suggested that mitophagy is also protective in this model [19]. Removing damaged mitochondria by mitophagy is a protective mechanism against alcohol-induced liver injury and steatosis because it serves to maintain a healthy population of mitochondria, which prevents cell death by reducing oxidative stress and preserving respiratory chain function and mitochondrial bioenergetics for efficient energy production. Mitophagy also helps prevent lipid accumulation in the liver by maintaining a healthy population of mitochondria capable of performing β-oxidation. Therefore, targeting removal of damaged mitochondria may be an effective therapeutic option for preventing progression of ALD.

Mitophagy protects against alcohol-induced liver injury by preventing cell death. Hepatocellular apoptosis in the liver plays a major role in progression of ALD [75,76,77], and mitophagy protects against alcohol-induced hepatocellular apoptosis and liver injury by removing damaged mitochondria [13,18,19]. As previously mentioned, alcohol metabolism produces ROS in the liver. In addition, mitochondria that are damaged by alcohol produce ROS and release pro-apoptotic proteins. Therefore, removal of these damaged mitochondria is necessary to reduce hepatocellular death and liver injury caused by heavy alcohol consumption [78].

Mitophagy also protects against alcohol-induced liver injury and cell death by maintaining mitochondrial bioenergetics for efficient ATP production by removing uncoupled mitochondria, which do not couple oxidative respiration to ATP production. Mitochondrial uncoupling causes damaged mitochondria and cell death by apoptosis in ALD. The reserve capacity of a mitochondrion, which is the cell energetic status or maximal energy capacity of a mitochondrion within the cell, decreases after chronic alcohol consumption [79]. Therefore, degradation of uncoupled mitochondria prevents oxidative stress and cell death by maintaining a healthy population of mitochondria that can efficiently produce energy necessary for cell survival.

**Figure 1:**
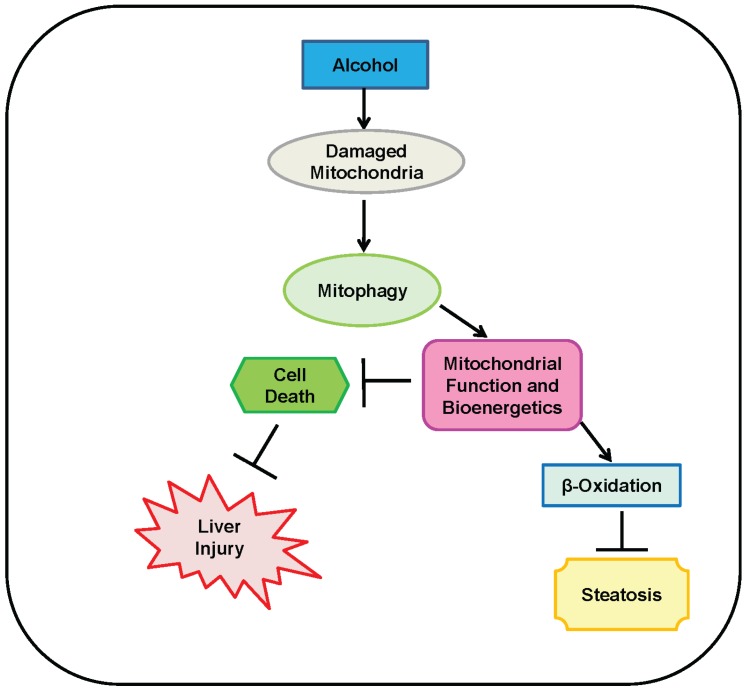
Mitophagy protects against alcohol-induced liver injury and steatosis. Alcohol initiates mitochondrial damage and dysfunction, which causes liver injury and steatosis. Mitophagy protects against liver injury and steatosis by removing these damaged and dysfunctional mitochondria, which maintains mitochondrial function and bioenergetics to avoid alcohol-induced cell death and liver injury. In addition, maintenance of a healthy mitochondria population by mitophagy allows for efficient β-oxidation, which reduces alcohol-induced liver steatosis. Two perpendicular lines represent “inhibition of”, and arrows represent “promotion of”.

Finally, mitophagy reduces alcohol-induced liver steatosis by preserving a healthy population of mitochondria capable of performing fatty acid β-oxidation. Heavy alcohol consumption inhibits β-oxidation, which results in an accumulation of lipids in the liver and development of liver steatosis [2,80]. Consequences of reduced or inhibited β-oxidation include accumulation of fatty acids that are esterified and stored as triglycerides, leading to fatty liver, or an accumulation of fatty acids that remain in an un-esterified form, which can be toxic to the liver and induce injury. In addition, faulty β-oxidation can decrease energy output [80]. Therefore, mitophagy helps defend against lipid accumulation in the liver by removing damaged mitochondria incapable of performing efficient β-oxidation. In addition to preventing fatty acid accumulation in the liver, β-oxidation in the liver is also important for sufficient energy production because it produces acetyl-CoA, which can enter the TCA cycle for ATP production [81,82]. A summary of mechanisms for protection against alcohol-induced liver injury and steatosis by mitophagy is shown in Figure 1.

Mitophagy is activated by many different cellular stress conditions including cellular ROS and mitochondrial depolarization [83], which are both caused by alcohol abuse [47,84,85]. Alcohol-induced ROS production depends on alcohol metabolism, which increases the ratio of NADH to NAD^+^. The increased NADH/NAD^+^ ratio promotes an excess flow of electrons into the mitochondrial respiratory chain, resulting in accumulation and leakage of electrons at the mitochondrial respiratory chain complexes I and III and subsequent production of ROS [47]. Alcohol exposure causes mitochondrial depolarization by damaging mitochondrial DNA and ribosomes, which leads to reduced numbers of mitochondrial proteins and decreased ATP synthesis [84]. Mitochondrial depolarization caused by alcohol is also dependent on its metabolism [85]. Mitophagy can also be induced my mitochondrial DNA damage. Mitochondria containing cytochrome oxidase subunit I mutant mitochondrial DNA are specifically selected for degradation by mitophagy [86]. In addition, treatment with the autophagy inducer rapamycin encouraged degradation of mitochondria containing a G11778A mitochondrial DNA mutation by mitophagy [87]. Futhermore, mitochondrial DNA mutations that lead to mitochondrial depolarization may also induce mitophagy. However, not all mitochondrial DNA mutations cause mitophagy induction [87]. ROS, mitochondrial depolarization, and mitochondrial DNA damage are all associated with ALD pathogenesis [88,89,90]. Therefore, any of these consequences of alcohol abuse may lead to mitophagy activation as an adaptive protective mechanism in the liver [91], but mechanisms for alcohol-induced mitophagy activation in the liver are not currently known. Mechanisms of mitophagy induction have been well studied in yeast and in mammalian cells, but most have not been studied in the liver. The E3 ubiquitin ligase Parkin is required for mitophagy induction in *in vitro* models, but several Parkin-independent pathways for mitophagy induction also exist. Parkin-dependent and independent mechanisms for regulation of mitophagy may both contribute to removal of damaged mitochondria and protection against alcohol-induced liver injury. Parkin-dependent and independent mechanisms for mitophagy induction are further discussed below.

## 5. Parkin-Dependent Mitophagy

Parkin is an evolutionarily conserved E3 ubiquitin ligase [92] encoded by the *Park2* gene [93] that has been shown to be required for mitophagy induction in *in vitro* models [40,94,95]. Parkin is recruited to damaged mitochondria by phosphatase and tensin homolog-induced putative kinase 1 (PINK1) to initiate ubiquitination of mitochondrial outer membrane proteins and subsequent mitochondrial degradation by mitophagy [96,97,98]. Parkin is well known for its protective role in the brain because loss of Parkin plays a role in development of Autosomal Recessive Parkinson’s disease, and the *Park2* gene was discovered in 1997 by Mizuno’s group as an unidentified gene responsible for this disease [99]. Even though the majority of research regarding Parkin is related to Parkinson’s disease, Parkin is also highly expressed in the liver in mice [20].

Parkin is well known to induce mitophagy in *in vitro* systems after treatment with the mitochondrial uncoupler carbonyl cyanide *m*-chlorophenyl hydrazone (CCCP) via ubiquitination of outer mitochondrial membrane proteins. Parkin was originally found to be involved in the mammalian mitophagy pathway in 2008 by Richard Youle’s group [95]. Parkin-dependent mitophagy requires both Parkin and PINK1. In healthy mitochondria, PINK1 is cleaved by presenilin associated, rhomboid-like (PARL) protein and then degraded by mitochondrial peptidases [100]. During mitochondrial depolarization, PINK1 is stabilized on the outer mitochondrial membrane [100], and it promotes Parkin-mediated mitophagy by recruiting Parkin to damaged mitochondria and activating Parkin’s E3 ligase activity via phosphorylation of Ser65 within Parkin’s ubiquitin-like (UBL) domain [101,102,103]. Once recruited to a mitochondrion, Parkin initiates the degradation of certain outer mitochondrial membrane proteins via the proteasome and recruits autophagy receptor proteins such as p62/SQSTM1. The autophagy receptor proteins then further recruit autophagosomes likely via directly interacting with the LC3 protein to selectively envelop the receptor decorated mitochondria and eventually transport the damaged mitochondria to lysosomes for their degradation. In addition to phosphorylating Parkin, PINK1 also phosphorylates ubiquitin at Ser65, which enhances Parkin-induced ubiquitination of substrate proteins on the outer mitochondrial membrane [103,104,105,106]. Parkin performs both lysine48 and lysine63 ubiquitination of several mitochondrial outer membrane proteins [94], and there are four known E2 ligases that interact with Parkin for mitochondrial protein ubiquitination: UBE2L3, UBE2D2, UBE2D3, and UBE2N [107]. Harper’s group found 36 outer mitochondrial membrane proteins that are Parkin substrates [108]. The most studied of these substrates for mitophagy initiation include the mitochondrial fusion proteins mitofusin 1 and mitofusin 2 (MFN1/2), the mitochondrial trafficking protein Miro1, translocase of outer mitochondrial membrane 20 (TOM20), and voltage-dependent anion channel (VDAC).

Ubiquitination of MFN1/2 leads to their degradation by the proteasome, which causes mitochondrial fission and fragmentation [94,109,110,111]. Mitochondrial fission is important for mitophagy induction in liver hepatocytes and other cell types because it allows segregation of damaged mitochondria from healthy mitochondria [20,35,112]. Mitochondrial fragmentation is also important for mitophagy initiation because smaller pieces of mitochondria can be more easily engulfed by autophagosomes [20,94,113]. MFN2 may also have a role in mediating Parkin recruitment to damaged mitochondria in addition to PINK1 [114]. Parkin-induced ubiquitination of Miro1 segregates damaged mitochondria from healthy mitochondria by initiating mitochondrial arrest, or inhibition of mitochondrial motility. Parkin-induced Miro1 ubiquitination was suggested to isolate a depolarized mitochondrion from the healthy mitochondria population to make it more easily targeted for engulfment by autophagosomes [115]. Parkin-induced ubiquitination of VDAC leads to recruitment of the autophagy adaptor protein p62/SQSTM1 to the mitochondria [110]. p62/SQSTM1 contains both a ubiquitin-associated domain (UBA) and a microtubule-associated protein light chain 3 (LC3)-interacting region (LIR), which allows it to bind to ubiquitinated proteins and organelles and transport them to autophagosomes for degradation by binding to LC3 on the autophagosome membrane [116]. The role of p62/SQSTM1 in mitophagy is currently controversial because some have shown it is required for mitophagy [110,117], while others have shown that it is not [118,119]. However, BCL2/adenovirus E1B 19 kDa interacting protein 3-like (BNIP3L), also known as Nix, is another Parkin substrate, and Parkin-mediated ubiquitination of Nix recruits the autophagy adaptor protein Neighbor of BRCA Gene 1 (NBR1) to damaged mitochondria to help shuttle them to the autophagosome for degradation [120]. Therefore, NBR1 and/or p62/SQSTM1 may act in the mitophagy pathway to degrade damaged mitochondria. However, the exact mechanism for autophagosome recruitment to damaged mitochondria after Parkin-induced ubiquitination of outer mitochondrial membrane proteins remains to be further studied.

Other proteins involved in activation of the Parkin-mediated mitophagy pathway are Nix and Smurf1. In addition to its role in recruiting NBR1, Nix also promotes the recruitment of Parkin to depolarized mitochondria after CCCP treatment in MEF cells. CCCP treatment causes mitochondrial depolarization, ROS accumulation, and translocation of Parkin to mitochondria in Parkin-overexpressing MEF cells while Nix-deficient MEF cells are resistant to mitochondrial depolarization and Parkin recruitment, suggesting that Nix may also play a role for Parkin-dependent mitophagy induction [117]. Smurf1 is an E3 ubiquitin ligase similar to Parkin that has a role in CCCP-induced mitophagy mediated by Parkin. Smurf1 does not initiate mitophagy, but its C2 domain is required for engulfment of damaged mitochondria by autophagosomes. The important role of Smurf1 in mitophagy is demonstrated by an accumulation of damaged mitochondria in heart, brain, and liver from Smurf1-deficient mice [121]. Parkin-induced mitophagy is also negatively regulated by ubiquitin-specific peptidases 30 (USP30) and USP15, which are deubiquitinases localized to mitochondria that inhibit Parkin-mediated mitophagy by removing ubiquitin from damaged mitochondria previously attached by Parkin [122,123].

## 6. Role of Parkin and Parkin-Dependent Mitophagy in Protection against Alcohol-Induced Liver Injury and Steatosis in Mice

As previously discussed, autophagy is protective against alcohol-induced liver injury and steatosis by removing lipid droplets and mitochondria by lipophagy and mitophagy, respectively [3,13,14,18]. Parkin has also been suggested to have other roles in maintaining mitochondrial function in addition to mitophagy because Parkin KO mouse brain mitochondria have decreased mitochondrial respiration along with altered levels of proteins involved in energy metabolism, protection from oxidative stress, and respiration [124,125,126]. In addition, the PINK1-Parkin pathway is involved in ethanol-induced hepatic mitophagy in rats because PINK1 is overexpressed in damaged mitochondria, mitophagosomes and perinuclear aggregations of damaged mitochondria in hepatocytes of ethanol-treated rats, which is supported by localization of LC3 to mitophagosomes after alcohol treatment [127,128]. Therefore, we investigated the role of Parkin as a protector against alcohol-induced liver injury and steatosis using two alcohol models: acute-binge [3,18,39] and Gao-binge (chronic plus acute-binge) [3,39,129]. The acute-binge model is designed to represent human binge drinking, and it is useful for studying initial stages of ALD such as steatosis, oxidative stress, and mitochondrial damage and dysfunction. The acute-binge model also causes mild liver injury [130,131]. The purpose of the Gao-binge model is to more accurately reflect human alcohol abusers because most people that chronically abuse alcohol also binge drink. In addition, the Gao-binge alcohol model causes greater liver injury, mitochondrial damage, oxidative stress, and steatosis compared to the acute-binge model [3,129,130,132]. Excitingly, Parkin protects against alcohol-induced liver injury and steatosis in mice after alcohol treatment by mitophagy activation. In addition, Parkin likely maintains mitochondrial function via other unknown mechanisms in addition to mitophagy after alcohol treatment [39]. Whole-body Parkin knockout (KO) mice have increased liver injury and steatosis after alcohol treatment compare to wild-type (WT) mice, which is likely due to the presence of severely damaged mitochondria in Parkin KO mouse livers after alcohol treatment, which are not present in WT mouse livers. Parkin KO mouse livers also have increased oxidative stress and decreased mitophagy and mitochondrial function compared to WT mouse livers after alcohol treatment resulting in greater mitochondrial damage and dysfunction in Parkin KO mouse livers compared to WT mouse livers [39]. Furthermore, WT mouse liver mitochondria are able to adapt to Gao-binge alcohol treatment while Parkin KO mouse liver mitochondria struggle to adapt to alcohol, as demonstrated by greater elongation of mitochondria in WT mouse livers than Parkin KO mouse livers after Gao-binge alcohol treatment [39]. Mitochondrial elongation is an adaptive mechanism to alcohol treatment [133]. While WT mouse livers have more elongated mitochondria after Gao-binge alcohol treatment, Parkin KO mouse livers have more swollen mitochondria [39]. In addition to mitophagy, Parkin also regulates mitochondrial biogenesis [134,135,136] and lipid transport [137]. It would be interesting to investigate these mitophagy-independent roles of Parkin in alcohol-induced liver injury and steatosis in the future. Overall, targeting Parkin and mitophagy may be a promising therapeutic approach for treatment of alcohol-induced liver disease.

Even though Parkin-induced mitophagy is activated and likely protects against alcohol-induced liver injury and steatosis in the liver, Parkin is not essential for mitophagy induction in the liver because mitophagy occurs in Parkin KO mice after treatment with alcohol [39]. Mitophagy also occurs in Parkin KO mouse livers after treatment with acetaminophen [138]. Mitophagy induction in Parkin KO mice is likely due to activation of adaptive mechanisms to compensate for the loss of Parkin. Even though mitophagy still occurs in Parkin KO mice after treatment with alcohol or acetaminophen, mitophagy levels are reduced in Parkin KO mice compared to WT mice [39,138]. Therefore, Parkin-independent mechanisms for mitophagy induction in the liver are likely not as efficient at inducing the mitophagy pathway as Parkin. However, Parkin-independent mechanisms of mitophagy induction in the liver are unknown and should be further explored in the future. Parkin-independent mechanisms for mitophagy induction are further discussed below.

## 7. Parkin-Independent Mitophagy

There are several Parkin-independent mediators of mitophagy that may be activated in the liver as an adaptive mechanism during chronic loss of Parkin including Nix/BNIP3L, Bcl2/adenovirus E1B 19 kDa protein-interacting protein 3 (BNIP3), Fun14 Domain containing 1 (FUNDC1), Cardiolipin, and Mitochondrial Ubiquitin Ligase 1 (Mul1) [20,38].

Mitophagy is induced during hypoxia by BNIP3, Nix, and FUNDC1. BNIP3 is a mitochondrial Bcl-2 homology 3 (BH3) domain-containing pro-apoptotic protein [139] that is expressed in liver, but it is not highly expressed under normal conditions [140,141,142]. Gene expression of BNIP3 is induced during hypoxia by hypoxia-inducing factor-1 alpha (HIF-1α), which binds to the HIF-1-response element (HRE) within the BNIP3 gene promoter [143]. Nix (BNIP3L) is a homologue of BNIP3 [144], and it activates autophagy by binding to Bcl-2 to dissociate the complex of Bcl-2 and Beclin-1, which is a protein necessary for initiation of autophagosome formation [145]. In addition, Nix interacts with the autophagosome membrane protein LC3 [146] to recruit autophagosomes to damaged mitochondria. Like BNIP3, expression of NIX is induced by HIF-1α during hypoxia [143]. NIX and BNIP3 seem to have complementary roles in mitophagy induction during hypoxia because individual loss of either BNIP3 or Nix does not affect autophagy, but combined loss of both NIX and BNIP3 inhibit hypoxia-induced mitophagy [145]. In addition to its role in mitophagy induction during hypoxia, Nix-induced mitophagy is also important for maturation of red blood cells, which destroy their mitochondria during the maturation process [147,148]. Furthermore, Nix induces mitophagy in cells undergoing high rates of oxidative phosphorylation by interacting with Rheb, which helps prevent mitochondrial damage and subsequent ROS production, mitochondrial dysfunction, cell death, and injury [149]. FUNDC1 is an outer mitochondrial membrane protein that also contributes to mitophagy induction under hypoxic conditions. However, the mechanism for FUNDC1 activation of mitophagy during hypoxia is different from BNIP3 and Nix because FUNDC1 expression during hypoxia is regulated by phosphorylation instead of transcription. In normal conditions, FUNDC1 is phosphorylated by the Src kinase on Tyr18 [150]. During hypoxia, the mitochondrial phosphatase phosphoglycerate mutase family member 5 (PGAM5) dephosphorylates FUNDC1 on Ser13 [151]. FUNDC1 contains an LC3-interacting region (LIR), allowing its interaction with LC3 on the autophagosome membrane, and the binding affinity of FUNDC1 for LC3 increases when FUNDC1 is dephosphorylated [150]. It is currently unknown which of these three genes (NIX, BNIP3, or FUNDC1) has the most important role in mitophagy induction during hypoxia. Alcohol induces hepatic expression of NIX and BNIP3, which are dependent on HIF and FoxO3a-mediated transcription [141,142]. FoxO3a KO mice have exacerbated acute alcohol-induced liver injury and steatosis [141]. However, whether there is a defect of mitophagy in FoxO3a KO mice after alcohol treatment needs to be further studied.

Cardiolipin is synthesized in the inner leaflet of the inner mitochondrial membrane [152]. Similar to Parkin, cardiolipin translocates to the outer mitochondrial membrane upon mitochondrial depolarization to induce mitophagy [153]. However, cardiolipin and Parkin seem to mediate mitophagy under different levels of mitochondrial depolarization. For example, CCCP-treated cells have PINK1 externalization to the outer mitochondrial membrane and recruitment of Parkin. However, rotenone treatment causes only approximately 10% of mitochondria to depolarize, leading to cardiolipin translocation to the outer mitochondrial membrane but not Parkin translocation. Cardiolipin translocates to the outer mitochondrial membrane after CCCP treatment, but not to the extent as during rotenone treatment, suggesting that there may be some overlap in Parkin and cardiolipin-induced mitophagy pathways or that they both may respond to CCCP treatment [154]. Interestingly, cardiolipin is also a pro-apoptotic protein, and post-translational modifications of cardiolipin influence whether it will induce cell death via apoptosis or cell survival through mitophagy. If cardiolipin on the outer mitochondrial membrane is peroxidized, it initiates apoptosis. Non-peroxidized cardiolipin present on the outer mitochondrial membrane induces mitophagy as a protective mechanism [153]. Cardiolipin may initiate apoptotic cell death if the mitophagy pathway fails to degrade damaged mitochondria [154]. Whether alcohol exposure can also cause cardiolipin translocation in hepatic mitochondria is currently unknown and requires further investigation.

The E3 ligase Mul1 was recently discovered to act in parallel to Parkin for mitophagy induction by ubiquitinating the outer mitochondrial membrane MFN1/2 proteins in *Drosophila* and mammalian cell lines during mitochondrial depolarization. Overexpression of Mul1 in *Drosophila* reverses Parkin/PINK1 mutant phenotypes including mitochondrial clumping and elongated mitochondria. In addition, PINK1 and Mul1 or Parkin and Mul1 double mutant flies have worsened phenotypes than either mutant alone including increased mortality and muscle degeneration, reduced levels of ATP, and damaged mitochondria. Furthermore, Parkin KO and Mul1 knockdown primary cortical neurons have increased mitochondrial depolarization, but neurons from Parkin KO mice with Mul1 knocked-down have greater increases in mitochondrial depolarization and neuron degeneration. Mul1 acts in a pathway independent of Parkin because knockdown or overexpression of Mul1 in Parkin-expressing HeLa cells does not affect Parkin translocation to mitochondria following mitochondrial depolarization [155]. Therefore, Mul1 may be an important compensatory pathway during loss or inactivation of Parkin.

Any of these Parkin-independent mediators of mitophagy may be responsible for compensatory mitophagy induction in Parkin KO mice after alcohol treatment. For example, BNIP3, Nix, or FUNDC1 may mediate mitophagy after alcohol treatment in the absence of Parkin because alcohol causes hypoxia in the liver and increases expression of BNIP3 and NIX [141,142,156,157,158]. Mul1 or cardiolipin may also have a role in mitophagy induction in Parkin KO mice after alcohol treatment because alcohol induces mitochondrial depolarization [85]. It would be interesting to determine other mediators of mitophagy in the liver after alcohol treatment in the future. Parkin KO mice with any of these other mitophagy mediators knocked down may provide evidence for one of these pathways acting in the absence of Parkin in the liver.

## 8. Mitochondrial Spheroids May Be a Novel Mechanism of Protection against Alcohol-Induced Liver Injury

In addition to mitophagy, mitochondrial spheroids may provide a novel mechanism of protection against alcohol-induced liver injury because they are induced as a stress response when mitophagy is impaired [40,42,159]. Mitochondrial spheroids are mitochondria that are shaped with a ring or cup-like morphology that can enwrap cytosolic contents such as other mitochondria, lipid droplets, or endoplasmic reticulum. They look similar to autophagosomes, but unlike autophagosomes, they have a small opening that connects their lumen to the cytosol. They may be able to degrade their enwrapped contents because they are positive for lysosome markers, but their actual ability to degrade their contents is unknown. Formation of mitochondrial spheroids requires ROS in addition to the mitochondrial fusion proteins MFN1 and MFN2, and deletion of either MFN1 or MFN2 inhibits mitochondrial spheroid formation. Therefore, Parkin inhibits mitochondrial spheroid formation by initiating degradation of MFN1 and MFN2 [40,41,42,160].

Acute-binge alcohol treatment causes increased levels of MFN1 and MFN2 and subsequent formation of mitochondrial spheroids in Farnesoid x receptor (FXR) KO mouse livers, but not in WT mouse livers [159]. FXR is the master regulator of bile acid homeostasis [161], and FXR KO mice have elevated levels of bile acids in their livers [162]. Autophagy is impaired by elevated bile acids in FXR KO mouse livers [163], and FXR KO mice have increased liver injury and steatosis after alcohol treatment compared to WT mice, which is likely due to an inability of FXR KO mice to remove damaged mitochondria and lipid droplets after alcohol treatment because of impaired autophagy [159]. Therefore, induction of mitochondrial spheroid formation in FXR KO mice may be an adaptive mitochondrial quality control mechanism for protection against alcohol-induced liver injury in the absence or down-regulation of mitophagy. Formation of mitochondrial spheroids, however, does not seem to be an efficient mechanism for reducing liver injury initiated by damaged mitochondria because even though FXR KO mice have induction of mitochondrial spheroid formation after alcohol treatment, they still have more alcohol-induced liver injury than WT mice. In addition, the physiological role of mitochondrial spheroid formation in the liver is not clear. Mitochondrial spheroid formation may represent damaged mitochondria or mitochondrial turnover, but the presence of lysosome markers on mitochondrial spheroids suggests that they have a role in turnover of mitochondria. However, whether or not these spheroids represent a protective mechanism against alcohol-induced liver injury is unknown, and our theory regarding this is speculative at this moment. The role of mitochondrial spheroid formation in alcohol-induced liver injury and steatosis requires further investigation.

**Figure 2:**
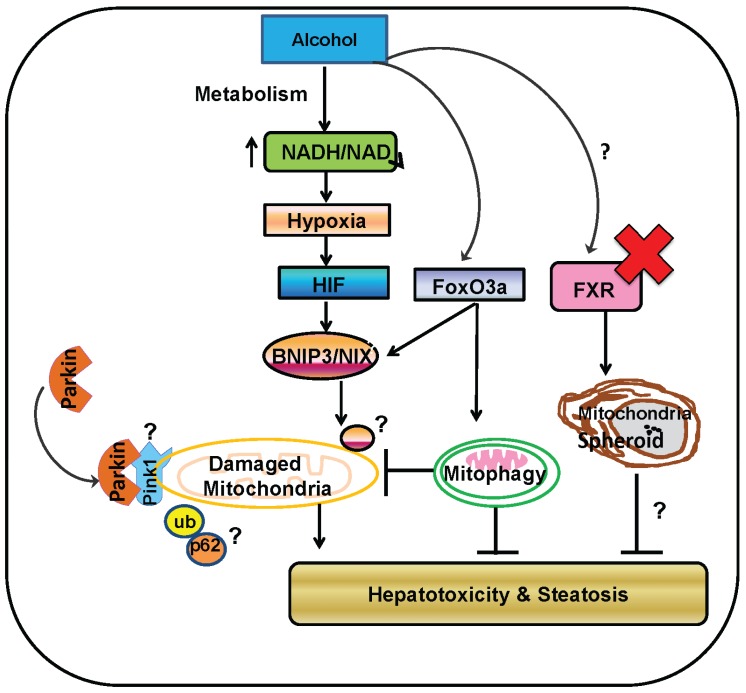
A proposed model for Parkin-dependent and-independent mitophagy and mitochondrial spheroid formation in alcohol-induced mitochondrial damage and liver injury. In ethanol-exposed hepatocytes, ethanol is metabolized by hepatic enzymes that lead to an increased NADH/NAD^+^ ratio and subsequent mitochondrial damage. Ethanol induces Parkin translocation from cytosol to a depolarized mitochondrion, which is likely mediated by stabilization of the mitochondrial protein kinase PINK1 on the depolarized mitochondrion. Parkin then ubiquitinates outer mitochondrial membrane proteins, which further recruits autophagy receptor proteins such as p62 to the damaged mitochondrion and triggers selective mitophagy. Meanwhile, ethanol also activates HIF and FoxO3a transcription factors resulting in increased expression of the two atypical BH-3 domain containing proteins BNIP3 and NIX, which might also serve as selective mitophagy receptors by directly interacting with the autophagy protein LC3 to recruit autophagosomes to damaged mitochondria independent of Parkin. In the absence of the nuclear receptor FXR, ethanol also induces formation of mitochondrial spheroids, which may also serve as an alternative mitochondrial quality control pathway to protect against alcohol-induced liver injury. In general, mitophagy protects against alcohol-induced liver injury and steatosis by removing damaged mitochondria and improving adaptive capacity of mitochondria to stress. Modulating Parkin-dependent and-independent mitophagy may be a promising avenue for preventing and treating ALD.

## 9. Concluding Remarks

ALD is a significant health problem worldwide, and there are currently no successful therapeutic options for alcohol-induced liver disease other than liver transplantation. Alcohol abuse is well known to cause mitochondrial damage, and mitochondria have many important roles in maintaining cellular homeostasis and survival. Mitophagy is an important pathway that helps maintain a population of healthy mitochondria by removing damaged mitochondria to prevent cell death and tissue injury. The most studied mechanism of mitophagy induction is Parkin-induced mitophagy, and Parkin protects against development of alcohol-induced liver injury and steatosis via initiation of mitophagy and maintenance of mitochondrial function [39]. Therefore, modulating Parkin and mitophagy may be a promising therapeutic approach for targeting drug and alcohol-induced liver injuries. Interestingly, mitophagy still occurs, although to a lesser extent compared to WT mice, in Parkin KO mice after alcohol and acetaminophen treatments [39,138], suggesting that adaptive compensatory mechanisms for mitophagy induction exist in the liver during chronic loss of Parkin. Several other Parkin-independent mechanisms for induction of mitophagy are surfacing including mitophagy mediated by BNIP3, Nix, FUNDC1, cardiolipin, and Mul1 that should be further investigated in the liver. Formation of mitochondrial spheroids may also be a novel mechanism of protection against alcohol-induced liver injury [159] and should be further explored. The possible molecular events for Parkin-dependent and-independent mitophagy and mitochondrial spheroid formation in regulating alcohol-induced mitochondrial damage and liver injury are summarized in Figure 2. A better understanding of mitophagy mechanisms and the purpose of mitochondrial spheroid formation in the liver may allow for future development of novel therapeutic options for treatment of ALD along with other forms of liver injury that involve mitochondrial damage and dysfunction.

## Abbreviations

ALD	Alcoholic Liver Disease
HCC	hepatocellular carcinoma
TNFα	tumor necrosis factor alpha
ADH	alcohol dehydrogenase
CYP2E1	Cytochrome p450 2E1
ALDH	aldehyde dehydrogenase
ROS	reactive oxygen species
FAEE	fatty acid ethyl ester
PINK1	phosphatase and tensin homolog-induced putative kinase 1
CCCP	carbonyl cyanide m-chlorophenyl hydrazine
PARL	presenilin-associated rhomboid-like
UBL	ubiquitin-like
MFN1/2	mitofusin 1 and 2
TOM20	translocase of outer mitochondrial membrane 20
VDAC	voltage-dependent anion channel
UBA	ubiquitin-associated domain
LC3	microtubule-associated protein light chain 3
LIR	LC3 interacting region
BNIP3L	BCL2/adenovirus E1B 19 kDa interaction protein 3-like
NBR1	neighbor of BRCA gene 1
USP	ubiquitin-specific peptidase
KO	knock out
WT	wild-type
BNIP3	BCL2/adenovirus E1B 19 kDa interaction protein 3
FUNDC1	Fun14 domain containing-1
Mul1	mitochondrial ubiquitin ligase 1
BH3	Bcl-2 homology 3
Hif-1α	hypoxia inducing factor alpha
HRE	Hif-1 response element
PGAM5	phosphoglycerate mutase family member 5
FXR	Farnesoid X receptor
